# Perceived sustainability of psychosocial treatment in low- and middle-income countries in South-Eastern Europe

**DOI:** 10.1192/bjo.2022.539

**Published:** 2022-08-15

**Authors:** Emina Ribic, Hana Sikira, Alma Dzubur Kulenovic, Tamara Pemovska, Manuela Russo, Nikolina Jovanovic, Tamara Radojicic, Selman Repisti, Miloš Milutinović, Biljana Blazevska, Jon Konjufca, Fjolla Ramadani, Stefan Jerotic, Bojana Savic

**Affiliations:** Clinical Center University of Sarajevo, Sarajevo, Bosnia and Herzegovina; Unit for Social and Community Psychiatry, Queen Mary University of London, London, UK; and WHO Collaborating Centre for Mental Health Services Development, Bart's and The London School of Medicine and Dentistry, Queen Mary University of London, London, UK; Unit for Social and Community Psychiatry, Queen Mary University of London, London, UK; Unit for Social and Community Psychiatry, Queen Mary University of London, London, UK; and Newham Centre for Mental Health, London, UK; Clinical Centre of Montenegro, Podgorica, Montenegro; University Clinical Centre Mother Teresa Skopje, Skopje, Macedonia; Department of Psychology, University of Prishtina ‘Hasan Prishtina’, Prishtina, Kosovo, Albania; and University of Prishtina, Kosovska Mitrovica, Serbia; Department of Psychology, University of Prishtina ‘Hasan Prishtina’, Prishtina, Kosovo, Albania; Department of Psychiatry, University Clinical Center of Serbia, Belgrade, Serbia; and Department of Psychiatry, Clinic for Psychiatry, Clinical Centre of Serbia, Belgrade, Serbia; University Clinical Center of Serbia, Belgrade, Serbia

**Keywords:** Schizophrenia, qualitative research, low- and middle-income countries, bipolar affective disorders, psychosocial interventions

## Abstract

**Background:**

DIALOG+ is an evidence-based, generic, cost-saving and easily deliverable psychosocial intervention, adaptable to clinicians’ personal manner of interaction with patients. It was implemented in mental health services in five low- and middle-income countries in South-Eastern Europe during a 12-month randomised-controlled trial (IMPULSE) to improve the effectiveness of out-patient treatment for people with psychotic disorders.

**Aims:**

To investigate barriers and facilitators to the perceived sustainability of DIALOG+ that has been successfully implemented as a part of the IMPULSE project.

**Method:**

Three months after the IMPULSE trial's end, perceived sustainability of the DIALOG+ intervention was assessed via a short survey of clinicians and patients who took part in the trial. Quantitative data collected from the survey were analysed using descriptive statistics; content analysis assessed qualitative survey data. The views and experiences of key informants (patients, clinicians and healthcare policy influencers) regarding the sustainability and scale-up of DIALOG+ were further explored through semi-structured interviews. These data were explored using framework analysis.

**Results:**

Clinicians mostly appreciated the comprehensiveness of DIALOG+, and patients described DIALOG+ meetings as empowering and motivating. The barrier most commonly identified by key informants was availability of financial resources; the most important facilitators were the clinically relevant structure and comprehensiveness of the DIALOG+ intervention.

**Conclusions:**

Participants showed a willingness to sustain the implementation of DIALOG+. It is important to maintain collaboration with healthcare policy influencers to improve implementation of DIALOG+ across different levels of healthcare systems and ensure availability of resources for implementing psychosocial interventions such as DIALOG+.

A number of studies have examined the implementation of evidence-based interventions in healthcare settings.^1^ Less research has been conducted into what happens after these implementation studies have ended, in other words, the intervention's long-term sustainability. There are numerous definitions of the term ‘sustainability’. Stirman and colleagues^2^ suggest that an intervention can be considered sustained if core elements are maintained after the initial implementation support has been withdrawn. Maintained core elements in this context refer to the intervention remaining recognisable or delivered at a certain level of fidelity to achieve the desired health outcomes. There is increasing recognition that the extent to which new interventions are sustained is influenced by many different factors and that there is a need to better understand what these factors are and their interaction.^3^

The IMPULSE project (Implementation of an effective and cost-effective intervention for patients with psychotic disorders in low- and middle-income countries in South Eastern Europe) was designed to improve the effectiveness of out-patient treatment for people with psychotic disorders. A type 2 hybrid pragmatic trial was conducted as part of the project, which simultaneously evaluated the effectiveness and implementation of DIALOG+, a psychosocial intervention.^4,5^ DIALOG+ is an evidence-based, generic, cost-saving and easily deliverable psychosocial intervention that is adaptable to clinicians’ personal manner of interaction with patients (http://impulse.qmul.ac.uk/dialog/).^6^ It is designed to be used during regular meetings between clinicians and patients. Aimed at reducing psychiatric symptoms and improving the quality of life of people with severe mental illness, it uses a solution-focused, technology-assisted approach. DIALOG+ has previously been shown to be effective in reducing clinical symptoms and improving quality of life in people with psychosis in the UK.^6^

DIALOG+ involves mental health professionals using a tablet computer to ask patients about their satisfaction with different life and treatment domains (mental health, physical health, accommodation, job situation, leisure activities, friendships, relationship with family/partner, personal safety, practical help, psychological help and medication). Patient and healthcare professional then work together to find solutions to concerns that are raised using a four-step process based on solution-focused therapy. This process is repeated during clinical appointments over several months. The DIALOG+ app is available for free download.^5^

During the IMPULSE trial, DIALOG+ was implemented in five low- and middle-income countries (LMICs) in South-Eastern Europe (SEE): Bosnia and Herzegovina, Kosovo (UN resolution), Montenegro, North Macedonia, and Serbia. In contrast to the well-organised support systems for psychiatric patients in high-income countries, the healthcare systems of these countries share many common unfavourable characteristics due to their similar socioeconomic and political backgrounds^7^ that differ from high-income countries: attitudes towards mental disorders, education, knowledge and skills of mental health professionals, as well as the levels of supervision and support that are available. Owing to its effectiveness and cost-effectiveness, DIALOG+ implementation could be an efficient solution to improve standards of care in LMICs.

Despite the fact that several quantitative studies suggest the benefit of psychosocial interventions on different mental health outcomes in LMICs, the quality of evidence is weakened by heterogeneity of outcomes and small sample sizes.^8^ A highly suggestive strength of association was found for adults with schizophrenia and depression in the general population; evaluating specific interventions, the most compelling evidence was found for cognitive–behavioural therapy and interpersonal therapy.^9^

Previous qualitative research exploring the experiences of participating in resource-oriented interventions is also limited and restricted to experiences of participating in single interventions,^10^ as opposed to exploring common processes across different resource-oriented interventions.

To explore pragmatic ideas about sustainability in the context of LMICs, this study aimed to investigate barriers and facilitators to the perceived sustainability of DIALOG+ that has been successfully implemented as a part of IMPULSE project. The assessment of perceived sustainability of DIALOG+ was done at both the individual and organisational level.

## Method

### Study design

We used a mixed-methods approach, involving primary qualitative and quantitative data collection methods, to explore perceived sustainability at both the individual and organisational level from the perspective of people with psychosis, clinicians and policymakers in mental health services in five SEE LMICs: Bosnia and Herzegovina, Kosovo, Montenegro, North Macedonia, and Serbia.

This study is based on data from the IMPULSE trial, which is a multi-country, pragmatic, hybrid effectiveness–implementation, cluster-randomised clinical trial involving 80 clinicians and 400 patients. Clusters were clinicians working with patients with psychosis, and each clinician delivered DIALOG+ to five of their patients. After patient baseline assessments, clinicians were randomly assigned to deliver either the DIALOG+ intervention or treatment as usual (TAU). The DIALOG+ intervention was delivered six times over 12 months during routine clinical meetings.

Data collected during and after the IMPULSE trial, but prior to awareness of trial outcomes, include transcripts, questionnaire responses, routinely collected monitoring data, and audio recordings of intervention and control sessions. Data analysis was descriptive and involved triangulation methods to compare findings across countries, stakeholder groups and data type.

Assessment of the perceived sustainability of DIALOG+ was conducted at both the individual and organisational level.

Sustainability at the individual level was assessed via a survey in two ways:

- clinician level: by recording numbers and characteristics of clinicians who were or were not interested in using DIALOG+ 3 months after the completion of the active implementation;
- patient level: by recording numbers and characteristics of patients who were or were not interested in using DIALOG+ 3 months after the completion of the active implementation.

Sustainability at the organisational level was assessed by conducting semi-structured interviews with key informants from all five participating countries. Key informants were defined as patients, clinicians and healthcare policy influencers.

### Participants

#### Individual-level sustainability

All trial clinicians involved in delivering the intervention (*n* = 40) and 97.5% of patients from the intervention arm of the trial (*n* = 202) responded to the survey.

#### Organisational-level sustainability

##### Patients

The research teams in each country identified between eight and ten patients from the intervention arm of the IMPULSE trial, with variations in age, gender, level of engagement with DIALOG+ and diagnosis. No more than two patients were selected from the same clinician (cluster).

In total, 40 individual in-depth interviews were conducted, transcribed and analysed. There were 21 men and 19 women; the average age of the informants was 43.20 years (s.d. = 10.04).

Of the 40 participants, 32 (80%) had a diagnosis of schizophrenia; the remaining 8 (20%) were diagnosed with bipolar disorder. There were nine informants from Serbia, seven from Montenegro, and eight each from Bosnia and Herzegovina, Kosovo and North Macedonia. Interviews were conducted in several different ways: 11 face-to-face, 21 by phone call and 8 by video call.

##### Clinicians

All clinicians from the intervention arm of the IMPULSE trial were invited to participate in the end-of-trial interviews. In total, 35 individual in-depth interviews were conducted, transcribed and analysed. Of these, 7 were with men and 28 were with women. The average age of the informants was 46.51 years (s.d. = 8.04); their average work experience in psychiatric institutions was 16.69 years (s.d. = 9.28). Of the 35 clinician participants, there were 19 psychiatrists, 11 nurses, 2 psychologists, 2 social workers and 1 psychiatry resident. There were four informants from Bosnia and Herzegovina, nine from Serbia, six from Montenegro and eight each from Kosovo and North Macedonia. Interviews with this sample were also conducted in several ways: 7 face-to-face, 26 by phone call and 2 by video call.

##### Healthcare policy influencers

The healthcare policy influencers were purposively sampled during the period from February to May 2020 to provide a variety of insights. Five teams (one from each participating country) identified three to five stakeholders from the following categories:

- directors/managers of mental healthcare institutions/services
- policymakers (e.g. Ministry of Health representatives)
- opinion makers (e.g. presidents/secretary-generals/board members of national psychiatric/psychological/nursing/social worker associations)
- presidents of patient and carer organisations
- service commissioners.

To ensure our findings are applicable and that they fit into different contexts, purposive variation sampling was used as participants were recruited from all five countries to obtain diverse perspectives on the topic. In total, 20 individual in-depth interviews were conducted, transcribed and analysed. The sample consisted of 10 men and 10 women. The average age of the informants was 50.3 years (s.d. = 9.95). There were four informants from the category ‘Directors/managers of mental healthcare institutions/services’, four from ‘Policymakers’, seven from ‘Opinion makers’, four from ‘Presidents of patient and carer organisations’ and one from the category ‘Service commissioners’. There were three informants from Bosnia and Herzegovina, five from Kosovo and four each from Montenegro, North Macedonia and Serbia. As these interviews were held at the beginning of the COVID-19 pandemic, all were conducted via video calls.

All participants received financial compensation for their time and effort in participating in the research.

### Procedure

Sustainability at the individual level was assessed using a simple survey: 3 months after the end of the IMPULSE trial, participants were asked to state whether or not they were willing to continue using the DIALOG+ intervention and to briefly explain their answer.

Regarding sustainability at the organisational level, topic guides for interviews with patients and clinicians were primarily designed for process evaluation of the IMPULSE trial. The purpose of completing a process evaluation of the IMPULSE trial was to contextualise the trial effectiveness and cost-effectiveness results, with respect to how the intervention may or may not have worked and what might be required for it to be sustained in clinical settings in LMICs.

Design of topic guides for interviews with healthcare policy influencers was substantially based on the dynamic sustainability framework.^11^ The topic guides were developed in English by a multidisciplinary team in an iterative process, and the draft versions were circulated among all collaborating centres for feedback. Discussions were held to ensure a unanimous understanding of the topic guide items.

The final versions of the topic guides for patients, clinicians and healthcare policy influencers (supplementary Appendices A, B and C, available at https://doi.org/10.1192/bjo.2022.539) were translated into the national SEE languages, prioritising the translation of the meaning of the topic guide items rather than word-for-word translations, so that the questions were formulated in everyday language and contextually sensitive.

### Data analysis

Survey data from the individual-level sustainability interviews were analysed using descriptive statistics. Data from open-ended questions including clinicians’ and patients’ reasons for deciding whether they wanted to continue using DIALOG+ were explored using content analysis.^11^ Two researchers (medical doctors with training in qualitative analysis) independently performed the analysis between January and March 2021; weekly meetings between the two were held to consensually develop and agree on the framework.

Survey data from the organisational-level sustainability interviews were explored using framework analysis, which is an analytical method involving highly structured coding of qualitative data into matrices of codes within an identified thematic framework in accordance with key issues and themes. The sample size was big enough to reach saturation point, according to the recommendation that the minimum sample size for adequate data saturation should be 13 participants.^12^

Two separate teams of researchers analysed data related to the organisational-level sustainability interviews (one team for the patient and clinician sample and another for the healthcare policy influencer sample); the corresponding author of this article was a part of both teams. The coding processes started with one researcher from each country translating one transcript into English and then sharing it with the rest of the team. The first 2 weeks of the analysis were reserved for becoming familiar with the content and discussion of ideas and themes that might emerge from the sample. Next, researchers started with open coding of the remaining transcripts. All the transcripts were coded in local languages.

The teams had weekly meetings from 15 June to 30 September 2020 so that researchers had the opportunity to discuss the coding process and consensually develop two frameworks: one based on the patient and clinician sample and another based on the healthcare policy influencer sample.

In the interviews with patients and clinicians, effectiveness of the intervention was assumed based on the original UK trial of DIALOG+^6^ because evidence from the IMPULSE trial was not yet available. Considering that the research team that conducted and coded the interviews had members from mixed backgrounds of research and clinical experience (medical doctors and psychologists), all IMPULSE researchers attended an intensive, internal qualitative research training programme led by the study coordinator team at Queen Mary University of London and learned how to carry out qualitative analysis. Researchers were not familiar with the interviewees before the start of IMPULSE project.

### Ethics

The authors assert that all procedures contributing to this work comply with the ethical standards of the relevant national and institutional committees on human experimentation and with the Helsinki Declaration of 1975, as revised in 2008. All procedures involving human participants/patients were approved by: the Ethics Committee of the Clinical Centre of the University of Sarajevo, Bosnia and Herzegovina (Ref.: 03-02-47500, date approved: 13 September 2018); the Ethical Professional Committee, Hospital and University Clinical Service of Kosovo, University Clinical Centre of Kosovo (Ref.: 904, date approved: 8 June 2018); the Ethical Committee for Research with Humans, Medical Faculty at the University of Cyril and Methodius in Skopje, North Macedonia (Ref.: 03-2237/12, date approved: 21 May 2018); the Ethics Committee for the Clinical Centre of Montenegro, Montenegro (Ref.: 03/01-11066/1, date approved: 19 July 2018); the Ethical Committee of the University of Belgrade Faculty of Medicine, Serbia (Ref.: 2650/V1-3, date approved: 26 June 2018). The local researchers provided detailed study information to potential participants and obtained written informed consent for research participation before data collection. Patient and clinician consent for the survey and interviews was obtained as part of consent procedures prior to the IMPULSE trial. Consent was given by all healthcare policy influencers before participating in the interviews.

## Results

### Individual-level sustainability

#### Clinician level

Of the 40 clinician respondents, 36 (90%) stated they would like to continue using the DIALOG+ intervention. Codes and categories organised into an analytical framework of reasons for continuing or not continuing are summarised in Table 1.

**Table 1:** Clinicians’ perceived sustainability of DIALOG+: analytical framework summary of categories and codes with their frequency of occurrence (*f*)

Positives						Negatives			
Positive attitude		DIALOG+ advantages		Perceived effectiveness		Negative attitude		Experienced burden	
Feasible	*f* = 9	Well-structured and comprehensive	*f* = 13	Patients are more proactive	*f* = 3	Negative feedback from patients	*f* = 2	Not practical	*f* = 1
General liking	*f* = 3	Better insight into patients’ condition	*f* = 6	Improves patients’ quality of life	*f* = 2	Thinks DIALOG+ is for younger clinicians	*f* = 2	Takes too much time	*f* = 1
Good for stable patients	*f* = 3	Facilitation of clinical work	*f* = 4	Patients see that clinicians care	*f* = 1	Not compatible with clinician's personality	*f* = 1		
		Improvement of therapeutic relationship	*f* = 3	Patients express themselves better	*f* = 1				
		Automatic and secure data storage	*f* = 3						
		Improvement of quality of sessions	*f* = 1						

The most frequently mentioned code was related to the good structure and comprehensiveness of DIALOG+, which was also the most commonly identified facilitator for reaching sustainability of this psychosocial intervention. Other facilitators included DIALOG+ providing better insight into patients’ condition and facilitation of clinical work and therapeutic relationships. Clinicians’ positive attitude towards DIALOG+ was mostly related to feasibility. Some clinicians also perceived DIALOG+ as effective, noting that patients became more proactive and that their quality of life was somewhat improved.

Clinicians who stated that they would not like to continue with the DIALOG+ intervention mentioned several reasons: some received negative feedback from patients and others thought that this intervention might be more suitable for younger clinicians. One clinician experienced the intervention as a burden, saying that DIALOG+ was not practical and that it required too much time to be suitable for routine sessions with patients.

#### Patient level

Of the 202 patient respondents, 148 (73.2%) stated that they would like to continue using the DIALOG+ intervention. Codes and categories organised into an analytical framework of reasons for continuing or not continuing are summarised in Table 2.

**Table 2:** Patients’ perceived sustainability of DIALOG+: analytical framework summary of categories and codes with their frequency of occurrence (*f*)

Positives								Negatives					
Positive attitude		Changes in treatment practice		Cognitive and behavioural changes		Implementation facilitators		Negative attitude		Experienced burden		Other	
General liking	*f* = 51	Wide range of topics	*f* = 15	Better insight	*f* = 9	Well- structured and comprehensive	*f* = 12	Prefers existing standard care	*f* = 9	Long sessions	*f* = 11	Relapse	*f* = 3
More time to express	*f* = 8	Monitor progress	*f* = 12	Improvement in organisation	*f* = 8	Good therapeutic relationship	*f* = 6	No specified reason	*f* = 8	Overwhelming	*f* = 10		
Higher frequency of sessions	*f* = 7	More time for discussion	*f* = 12	Solution oriented	*f* = 3			Repetitive	*f* = 1	Technology as a burden	*f* = 6		
Prefers novel methods	*f* = 6	More engaged clinician	*f* = 8	Sense of comfort	*f* = 5								
				Empowering	*f* = 8								

The most frequent code among patients who responded affirmatively to continuing to use DIALOG+ was ‘General liking’, commonly suggesting that DIALOG+ was beneficial for them. Patients also reported a positive attitude towards the more frequent sessions that they had with DIALOG+ in comparison with TAU.

The category ‘Changes in treatment practice’ explored differences that DIALOG+ brought to treatment options. Participants described DIALOG+-mediated meetings as longer and more detailed and also reported that clinicians were more engaged during DIALOG+ sessions, which they considered to be a factor that improved the therapeutic relationship.

Participants also reported cognitive and behavioural changes in relation to introducing DIALOG+, describing better insight into their condition, improved organisational skills and better structure in their everyday life.

Facilitators of the implementation of DIALOG+ included the good structure and comprehensiveness of the intervention. Participants often mentioned that an existing good therapeutic relationship with their clinician served as a foundation for getting used to novel aspects of the intervention.

Among 49 patients who responded negatively to continuation of use of the DIALOG+ intervention, a proportion reported that they simply prefer TAU over DIALOG+ and some were just no longer interested, without specifying any reason. One described DIALOG+ sessions as repetitive in the sense that the same questions were asked during every session, causing loss of interest in the intervention.

Some patients also experienced several burdens, describing DIALOG+ sessions as too long or overwhelming. A few reported technology as an experienced burden, suggesting that using the tablet was the problem, but that they liked the intervention itself. Three participants were in the relapse phase of their disorders at the time this survey took place, so they did not share their opinions with the research team.

### Sustainability at the organisational level

#### Barriers and facilitators to sustainability – clinicians’ and patients’ perspective

Framework analysis yielded two themes, which were interpreted as barriers and facilitators (Appendix 1 below).

Limited financial resources were identified as a barrier, with participants mostly discussing expenses related to purchasing tablet computers. The findings suggest that these expenses were not negligible, as both clinicians and patients shared the same thought:

‘ … but I still think it will be a great expense for our country to purchase tablets for every doctor, I do not know whether it will be possible’ (Patient, North Macedonia).

When discussing the lack of clinical staff, all clinicians pointed out that they were overloaded with the number of patients, which had a significant impact on their ability to deliver DIALOG+ to its full capacity. Some of the clinicians suggested that DIALOG+ should be delivered not only by psychiatrists, but also by other clinical staff included in the care of patients:

‘Well, I think that the only obstacle is that due to the volume of work, we are often limited in time […] doctors here can't dedicate so much time, for example, to one patient or a certain group and so on, but with more people it's feasible’ (Clinician, Montenegro).

Patients also expressed concerns about lack of clinical staff and their availability in general. Some patients were sceptical about clinicians’ capacity to deliver DIALOG+:

‘I don't know if there are enough rooms, enough doctors, if there is enough time and all other things, but I think it is possible’ (Patient, Serbia).

Another barrier was that implementation in a hospital-based setting was perceived as undesirable. Some of the clinicians thought that DIALOG+ meetings should instead be delivered in mental healthcare centres in the community:

‘I think they would utilise it most in mental health centres, in the primary healthcare system and so on […] Because it doesn't happen too often that patients come here to me specifically for some control session or conversation’ (Clinician, Bosnia and Herzegovina)

‘Sometimes it didn't suit them if they didn't live nearby, if they weren't from the city, but from somewhere around … they do not have an adequate means of transportation nor money to pay for transportation’ (Clinician, Serbia).

Most of the patients also reported the distance to clinical centres as a barrier, especially because it required additional time and travel costs. Some patients also reported that in-patient wards were not a good setting for delivering DIALOG+, especially regarding all the unpleasant memories the psychiatric ward could bring up:

‘It was a bit far for me to come to sessions, I live in another city, I don't know … if this was available in our local mental health centre, with our psychologist, it would be great’ (Patient, Bosnia and Herzegovina)

‘For example, I personally would not like it to be in the ward itself […] Because somehow those ugly pictures come back to a man, it's not nice to anyone there’ (Patient, Montenegro).

Another theme that emerged as a barrier was incompatibility with existing practice, which was recognised by both patients and clinicians. Some clinicians expressed regret for not having enough time to implement novel methods in their work, and they also said that it could be complicated to include DIALOG+ in already existing clinical protocols. Patients expressed the opinion that clinicians, particularly older ones, might not be able to manage the technology:

‘Our politics are bad and procedures hinder everything … I think you will come across so much procedural nonsense and it will bother you the most. Systemically, it would also be good to find a way for us professionals to engage, to learn new things’ (Clinician, Bosnia and Herzegovina)

‘ … computer literacy for doctors might be a problem, although they are all skilled in internet and in everything’ (Patient, North Macedonia).

Regarding sustainability and scale-up facilitators, five themes emerged. The first was patients’ ability to choose the treatment option:

‘Well, it wouldn't be bad to use it sometimes. I'm not always in the mood, but I wish I had the option to say, uh … to say whether I want to’ (Patient, Bosnia and Herzegovina).

The majority of participants expressed willingness to continue and scale up the intervention, which emerged as a theme itself. Most of the clinicians reported that they would continue using it for regular check-ups after the IMPULSE project was over:

‘[…] it would be curative and valuable for patients themselves to have these routine controls and conversations, enriched by such an intervention. I say that it would be really effective to become a part of any control, to implement it as it is, I think it would be positive’ (Clinician, Montenegro).

Most of the patients reported willingness to continue with DIALOG+ sessions because of their positive experiences with it. They also reported more control over their condition with DIALOG+:

‘Well, I would like it, I think that way I could see better if things are not going well, if my condition starts getting worse … I would be more in control of my illness’ (Patient, Bosnia and Herzegovina).

Another facilitator was the ability to task-shift to non-psychiatrists. This theme was recognised by both clinicians and patients, who saw it as an opportunity to include more people in mental healthcare services:

‘It doesn't have to be the sole responsibility of a psychiatrist. I think that other therapists should also be included, occupational therapists … ’ (Clinician, Serbia)

‘Maybe it would be good to train additional people for this, or for psychologists to do this with in-patients, they perform different tests anyway. Or … a nurse did this for me, why not? Like it has to be a doctor?’ (Patient, Bosnia and Herzegovina).

Many participants expressed that the DIALOG+ intervention should be registered as a mental health service, which would make it available and valued in practice:

‘Well, that's something that needs to go into the national strategies, right? Strategies, action plans and what would, something that would be introduced independently […] as a part of the guidelines of good clinical practice’ (Clinician, Montenegro).

Some clinicians suggested the intervention would be more suitable and beneficial for patients with affective disorders:

‘I think that D+ would have a much greater application and much greater efficacy in patients with neurotic disorders … because I believe that psychosocial support means much more to them than to patients with schizophrenia’ (Clinician, Serbia).

#### Barriers and facilitators to sustainability – healthcare policy influencers’ perspective

All identified codes, categories and themes were organised into an analytical framework, as summarised in Appendix 2 below.

The theme ‘Factors influencing sustainability’ refers to facilitators, barriers and other factors that stakeholders recognised as important for sustaining the DIALOG+ intervention:

‘Although in the beginning it turns out that you need a little extra time in the ambulatory work, however, if you look at things later, time needed decreases’ (Healthcare policy influencer, Serbia).

Nearly all the healthcare policy influencers talked about the availability of resources. Besides financial resources (the most frequent topic), participants discussed human resources as well, noting management's role as the most important one. In terms of anticipated barriers or obstacles, complex procedures were seen as one of the main barriers, mostly mentioned in relation to the process of including DIALOG+ in the official services list. Along with resistance of institutions and mental health professionals, procedural barriers were described in terms of institutional bureaucracy and the importance of policymakers’ attitude towards implementing novel interventions.

The findings suggest that the most important facilitators were the structure of DIALOG+, the influential role of the clinician in a well-established therapeutic relationship, and technology as a tool for advanced monitoring of the patient's condition and reduction of the extensive paperwork burden. The discussion about training options was highly based on models of training, where participants mostly talked about the peer-to-peer training model, as well as the importance of networking and supervision. The role of caregivers was mostly viewed through supporting and motivating patients during their participation in DIALOG+, although some of the key informants noticed that caregivers might be included in monitoring the patient as well.

The theme ‘Adaptability to context’ explored ideas about the fit of DIALOG+ into healthcare systems in LMICs, as well as its applicability for patients and clinicians in these countries:

‘I would say that young people, enthusiasts, they will adapt it for sure … it can be a nurse who wants to do it, so it depends on the person more than on the profile of the profession’ (Healthcare policy influencer, Bosnia and Herzegovina).

Most participants discussed the healthcare system, suggesting that implementing DIALOG+ might improve existing practices. Furthermore, they discussed the applicability of DIALOG+ and pointed out the similarity of DIALOG+ to existing practices, as well as its compatibility with them. Regarding the target level(s) of healthcare, most stakeholders agreed that DIALOG+ should be implemented at all levels of healthcare, since the intervention is not complicated and can be used by services which are not specialised. Informants mostly agreed that all levels of healthcare should be receptive to this approach, especially out-patient services. Regarding the profile of mental health professionals who are suitable for delivering DIALOG+, nearly all interviewees agreed that any healthcare professional would be able to deliver DIALOG+ after being trained and that the most important factor for selecting professionals should be their motivation, regardless of their age or profession (nurses, doctors or social workers).

The theme ‘Promotion’ explored potentially useful ideas for future promotional activities for DIALOG+:

‘If you show that DIALOG+ takes significantly less time, less training, costs less, and you expect to get a similar effect, then it seems extra-stimulating. When a comparison is made with some other methods, which are much more demanding and require more costs and more trained staff, and if you have a good effect there with less effort, I think that is a good way to present it’ (Healthcare policy influencer, Serbia)

‘At organised meetings between the people involved who use that program – nurses, doctors and clinicians – to make suggestions for its improvement, for its more efficient functioning’ (Healthcare policy influencer, North Macedonia).

Informants mostly addressed the importance of the IMPULSE trial results, particularly its proven effectiveness and cost-effectiveness, as the best promotion for DIALOG+. When referring to potential target groups for promotions, some informants stated that promotional activities should be focused on targeting professionals and policymakers at national and international conferences, whereas others thought it would be better to focus on potential users of the intervention in non-governmental organisation (NGO) users’ associations.

Finally, the theme ‘Anticipated impacts’ emerged as a set of ideas related to stakeholders’ views on potential effects that DIALOG+ might have on different groups of users:

‘Thus, if this approach, which I would call a technology-focused approach, is enabling good communication between the doctor and the patient, the main service is lessening of the level of symptoms and enhancing patient functioning. In addition, it improves the individual potential of people with mental disorders in their daily activities, by including social and job-related areas.’ (Healthcare policy influencer, Kosovo).

Among other topics, they discussed potential advancement of clinicians’ professional roles and the greater involvement of caregivers in treatment. However, most of the informants believed that DIALOG+ would affect patients the most, as the category ‘Impact on patients’ was mentioned twice as often as all other codes combined.

## Discussion

### Main findings

#### Individual-level sustainability

The majority of participants in the intervention arm reported willingness to continue using DIALOG+. The good structure and comprehensiveness of the intervention were also identified in a previously published article about DIALOG+.^13^ Reoccurrence of this finding across different studies is a promising result which implies that sustainability of DIALOG+ might be feasible owing to its practicality and completeness over a broad scope. Other identified facilitators, such as DIALOG+ providing better insight into patients’ conditions, facilitating clinical work and improving therapeutic relationships, were also previously described in literature.^14^ All these identified benefits of implementing DIALOG+, combined with the frequently mentioned feasibility of the intervention, represent favourable features in terms of achieving sustainability of DIALOG+. It is encouraging to see that clinicians perceived certain benefits of this intervention in patients, such as increased proactivity and improvement in quality of life. Furthermore, patients reported these comprehensive DIALOG+ meetings and active engagement to be comforting for them, making them less anxious between meetings. They appreciated having longer and more frequent sessions, thus having more time to express themselves, and considered that to be an improvement in the therapeutic relationship. These perceived benefits of DIALOG+ are encouraging to record in LMICs, where there is a lack of services and human resources in mental healthcare, and usually insufficient capacity to offer little beyond pharmacological prescribing.^15^

Regarding experienced burden, some clinicians felt that implementing DIALOG+ took too much time and they did not find it practical. Some patients also identified longer sessions as a burden; some of them reported that DIALOG+ might be overwhelming and tiring for them because of the many life domains that need to be discussed.

Several clinicians also noted that this intervention might be more suitable for younger colleagues, probably because they were more accustomed to using technology.^16^ Some patients also described the technology (tablets) as a burden since they were not familiar with it. The challenges of implementing effective digital behaviour change interventions are similar to those faced by other behaviour change interventions, including different levels of engagement with the intervention, defining measurements of effectiveness and cost-effectiveness, and complying with regulatory, security and ethical requirements.^17^ However, digital interventions offer many new opportunities, such as taking advantage of data they generate, their potential adaptability, increased reach and decreased costs.

#### Sustainability at the organisational level

Financial resources were viewed as one of the most important potential barriers for sustainability of DIALOG+ in LMICs. This finding emerged across all participant groups and was in accordance with the existing literature on intervention sustainability in LMICs.^14^ Furthermore, financial resources were often mentioned during discussion about other barriers, indicating that this barrier might be crucial in facilitating successful sustainability of DIALOG+.

It is also important to address which levels of healthcare systems in LMICs would be the most desirable for future implementation, as both clinicians and patients suggested that clinical centres (tertiary level) might not be the best option owing to extensive workload, accessibility problems and patients’ unpleasant memories of psychiatric wards in acute phases of their illness. According to available research,^18^ barriers related to implementation of new interventions in the hospital environment include increased staff workload, lack of time for implementation, staff shortages and lack of private space for interventions requiring sensitive discussion. Therefore, community mental health centres might offer a more suitable environment for DIALOG+, as they might be more easily accessible and more comfortable for a wider group of patients.^19^ However, it is important to mention that there is no strong evidence supporting either hospital-based care or community-based services in terms of providing a comprehensive system of mental healthcare – a necessary balance includes both hospital and community components.^20^

When it comes to the complex topic of lack of compatibility with existing healthcare systems, clinicians and patients described issues such as limited resources, labour shortage and stigma, as well as lack of supervision and available support. These issues are not easily manageable and need to be addressed strategically on national and international levels in LMICs.^21^ Furthermore, the idea of task-shifting,^22^ which is defined as training non-physician healthcare workers to deliver tasks that are conventionally performed by physicians, might be seen as a potential solution to limited healthcare access in LMICs.^23^ Several recent studies suggested that task-shifting in community-based settings not only improved access to mental health services, but also strengthened the rapport between implementers and the community and increased intervention acceptance and engagement.^24^

Regarding the potential resistance of mental health professionals to implementation of novel interventions, it takes both well-designed technological tools and motivated professionals to lay the groundwork for sustainable implementation of DIALOG+. However, skilled clinicians tend to be very good at overcoming barriers when they are provided with sufficient training and resources.^16^ Finally, patients’ ability to choose between DIALOG+ and other treatment options at the start of the session might be a valuable strategy and facilitator, as sudden changes in patients’ treatments can be harmful to the sustainability of this novel intervention.^25^

It is also important to consider potential benefits of DIALOG+ for people with other diagnoses beside psychotic disorders, such as affective disorders. It is possible that DIALOG+ might be more effective in people with anxiety or depression because they might have more utilisable personal and social resources than individuals with psychotic disorders.^26^ In a recently published study, authors found that DIALOG+ could be effective for improving quality of life and reducing psychiatric symptoms in people with depressive and anxiety disorders in LMICs.^27^

In terms of other anticipated barriers or obstacles, institutional bureaucracy was consistently discussed as a major challenge. The so-called command-and-control management approach^28^ in healthcare systems is a major barrier to implementation processes in any country, especially in LMICs. This was also noticeable in our sample, where several informants emphasised the importance of the attitude of certain policymakers or managers towards any kind of novel intervention. One of the possible solutions to this problem could be distributed leadership across levels and positions in a healthcare system working together to achieve collective goals,^29^ but that is likely to be a long-term objective.

The most important facilitators identified were the structure of DIALOG+, the influential role of clinicians in a well-established therapeutic relationship and technology as a tool for advanced monitoring of the patients’ condition and reduction of extensive paperwork. This can be easily interpreted in the context of the above-mentioned barriers and resources. Both well-structured psychosocial interventions and use of technology can save time. This is an important consideration in LMICs,^30^ where services are usually overburdened with patients and extensive paperwork, so that clinicians do not have much time for each patient. Of course, one cannot expect that implementation of DIALOG+ can solve all of the above-mentioned issues, but considering that routine clinical meetings are already part of standard care for patients in LMICs, incorporating DIALOG+ could improve the therapeutic effectiveness without any major changes to the organisation of routine consultations.^27^ Furthermore, the treatment in this context mostly relies on clinicians’ personal communication skills as a cheap yet very effective resource, so it is not surprising that the role of the clinician in the patient–clinician relationship was seen as such an important facilitator.

It is noticeable that the majority of stakeholders thought that including DIALOG+ in official service lists was a first step for its successful implementation and sustainability. To overcome barriers related to complex bureaucratic procedures, it is very important for managers to familiarise themselves with these procedures and collaborate with service commissioners.

### Strengths and limitations of the study

This study has several strengths. First, it used qualitative methodology to investigate sustainability of a digital mental health intervention in LMICs. Qualitative research is not widely used in this context and the concept of sustainability of mental health interventions is not commonly investigated in LMICs.^30^ Second, interviewing stakeholders with diverse backgrounds allowed for in-depth understanding of this complex phenomenon. Furthermore, researchers from all participating countries in the IMPULSE project were included in data collection and analysis, and all transcripts were coded in their native languages, which allowed a more comprehensive view and understanding of this cultural context and more accurate interpretation of the results. Finally, an independent researcher who did not collect data served as a quality check, and all research team members always aimed for reaching intersubjective agreement during the whole process of the analysis.

This study also has an important limitation that should be mentioned. Research activities after the end of the IMPULSE trial were limited owing to the COVID-19 pandemic, so actual sustainability of DIALOG+ was not properly explored, as routine sessions were cancelled from March 2020. Subsequently, it is possible that this paper might not provide robust indicators of the sustainability of the DIALOG+ intervention.

### Implications

#### Research implications

The DIALOG+ intervention was originally developed and implemented in secondary mental health services, but the analysis of sustainability at the organisational level shows that the informants frequently suggested implementation of DIALOG+ in primary healthcare. The informants mostly addressed the intervention's simplicity and structure, which can be widely utilised by professionals in primary healthcare. Furthermore, the primary level of healthcare in LMICs is where the intervention can possibly reach the widest population in terms of prevention and early treatment of mental disorders, as only the patients with more complex clinical conditions get to be treated at higher levels of the healthcare system. Altogether, more research is needed to explore barriers and facilitators for DIALOG+ in primary care.

#### Organisational implications

It is important to maintain collaboration between researchers, clinicians and healthcare policy influencers in terms of improving implementation of DIALOG+ across different levels of healthcare systems and ensuring availability of resources for implementing other psychosocial interventions similar to DIALOG+. This collaboration might help researchers and clinicians to more easily overcome the barriers related to the implementation process, and it might also provide healthcare policy influencers with reliable insight into practice settings, thus helping them with setting priorities in cost planning.

#### Clinical implications

Most of the patient participants showed a willingness to sustain the implementation of DIALOG+ in their treatment. Our findings suggested that the DIALOG+ intervention might be first offered to patients who have reached the remission phase of their illness, regardless of their primary diagnosis. However, it is also important to note that patients need to have adequate insight and cognition, as this intervention does require introspective abilities and certain cognitive efforts. Our findings also suggested that patients’ age should not be a determining factor for offering the DIALOG+ intervention.

In the matter of which healthcare professionals should deliver DIALOG+, our findings indicated that the most important factor is the individual's motivation for adopting this novel intervention, rather than their profession or status.

## Data Availability

The data that support the findings of this study are available on request from the corresponding author. The data are not publicly available owing to confidentiality policies.

## Supplementary material

For supplementary material accompanying this paper visit http://doi.org/10.1192/bjo.2022.539. click here to view supplementary material

## Appendices

### Appendix 1 Barriers and facilitators to sustainability and scale-up of DIALOG+: clinicians’ and patients’ perspectives

Theme 1 Sustainability and scale-up barriers:

1. Limited financial resources
2. Lack of clinical staff
3. Implementation in hospital-based setting perceived as undesirable
4. Lack of compatibility with existing practice

Theme 2 Sustainability and scale-up facilitators:

1. Ability to choose preferred treatment option
2. High willingness to continue using intervention and scale it up
3. Implementation in community-based care setting
4. Task-shifting to non-psychiatrists
5. Registration of intervention as an official mental health service
6. Intervention perceived as suitable for setting beyond healthcare and for other diagnoses.

### Appendix 2 Healthcare policy influencers’ perceived sustainability of DIALOG+: analytical framework summary of categories and themes

Theme 1 Factors influencing sustainability:

1. Resources
2. Anticipated barriers/obstacles
3. Facilitators
4. Role of caregivers

Theme 2 Adaptability to context:

1. Healthcare system: DIALOG+ fit
2. Patients: DIALOG+ fit

Theme 3 Promotion:

1. Demonstrating efficacy and effectiveness of DIALOG+
2. Target groups for DIALOG+ promotion

Theme 4 Anticipated impacts:

1. Systematic impacts of DIALOG+ implementation
2. Impacts on professional roles
3. Impacts on patients
4. Impacts on caregivers
5. Future possibilities.
