# Perceived sustainability of psychosocial treatment in low- and middle-income countries in South-Eastern Europe – CORRIGENDUM

**DOI:** 10.1192/bjo.2023.18

**Published:** 2023-08-04

**Authors:** Emina Ribic, Hana Sikira, Alma Dzubur Kulenovic, Tamara Pemovska, Manuela Russo, Nikolina Jovanovic, Tamara Radojicic, Selman Repisti, Miloš Milutinović, Biljana Blazevska, Jon Konjufca, Fjolla Ramadani, Stefan Jerotic, Bojana Savic

This article was published with an error in the affiliation of Biljana Blazevska Stoilkovska. She is in fact affiliated with the Department of Psychology, Faculty of Philosophy, Ss. Cyril and Methodius University in Skopje, Skopje, Republic of North Macedonia.
