# Supplementary material for: Perceived sustainability of psychosocial treatment in low- and middle-income countries in South-Eastern Europe
Source: BJPsych Open. 2022 Aug 15;8(5):e156. doi: 10.1192/bjo.2022.539 (PMC9438482; doi:10.1192/bjo.2022.539)
Supplement: Supplementary file 1 [file S2056472422005397sup001.docx]

# Appendix A- topic guide for in-depth semi-structured interviews with healthcare policy influencers

**TOPIC GUIDE FOR IN-DEPTH INTERVIEWS WITH KEY INFORMANTS**

Exploring sustainability of DIALOG+ intervention

**To explore ideas and potential changes in:**

- Sustainability of the DIALOG+ intervention
- Scaling up the intervention

**1) INTRODUCTION**

- Introduce yourself- *I am XXX. I am a researcher with the IMPULSE study which investigates implementation of DIALOG+ intervention. I will shortly introduce you to the study and preliminary findings.*
- Present the study using standardized presentation (info on DIALOG+ and IMPULSE trial).
- Explain the purpose of the interview-

**This intervention has been found effective and cost effective in the original trial conducted in the United Kingdom. It reduced patients’ symptoms, improved their quality of life and saved money for the healthcare system. At the moment DIALOG+ has been implemented in more than ten countries around the world. We are in process of completing a trial in ______________ (state your country).**

**In this interview I would like to find out more about your views on sustainability of the DIALOG+ intervention, assuming that the intervention will be effective and cost effective in _______________(state your country).**

- Provide assurances about confidentiality and emphasize that personal disclosures are not expected.
- Explain what happens to data collected – transcribing, reported, anonymity – check how they would like to be referred to during recording.
- Introduce tape recorder.
- Explain how interview works – interview will not say very much and will tend to ask questions, all views are important, no right or wrong answers, looking for a range of views.
- Explain they should say if they are feeling stressed/uncomfortable and that they do not have to answer a question and that a topic can be changed if they need to.
- Invite any questions.

**General probes to use during interview:**

- “Tell me more about that.”
- “What is it like?”
- “What led you to feel, think in that specific way... “
- “Can you think of another example of this?”
- “Give me more details please.”
- “I’d like to hear more.”

1. **WARM UP**

**In your opinion, how does DIALOG+ fit into existing health care services/programs in our country?**

Probes:

- On what level of heath care system would implementation of DIALOG+ be the most beneficial for patients (e.g. Primary level- community mental health centres, secondary level- hospitals, tertiary level- clinical centres)? Why?

1. **SUSTAINIBILITY**

**As part of this research, the DIALOG+ intervention has been implemented in two psychiatric services in our country ____________ (state their names). This means that we trained clinicians to deliver the intervention, bought computer tablets and supported clinicians to recruit and retain patients in the study. The study is about to end and there will be no additional support to deliver DIALOG+ intervention.**

**We are interested in your views on how to sustain the intervention after the end of study or once the research money is no longer available.**

**In your view, what needs to happen so that services and clinicians continue to provide the DIALOG+ intervention?**

Probes:

- How can DIALOG+ be included in the official list of provided services in these institutions?
- What is the role of clinical leadership for sustainability of this intervention?
- What type of support do you think is necessary for clinicians to continue providing DIALOG+ to patients? (e.g. protected time, training, supervision)
- How would you include DIALOG+ training, if at all, into clinicians’ continuous professional development?

**When it comes to patients, what needs to happen so that patients continue to participate in DIALOG+ sessions?**

Probes:

- What type of support do you think is necessary for patients to continue participating in DIALOG+?
- What do you think caregivers’ role should be in supporting patients’ participation in DIALOG+?

1. **SCALING-UP THE INTERVENTION**

**Now we would like to discuss topics around rolling-out the intervention beyond the current study sites. As mentioned, at the moment the intervention is being implemented in __________sites.**

**In your opinion what is necessary to happen for other health services/clinics to adopt the intervention across the country?**

Probes:

- Do you expect any difficulties with implementation of DIALOG+ according to standardized policies and regulations? If yes, could you tell more about that?
- Do you think that long-term implementation of DIALOG+ requires additional funds? Please elaborate your answer (e.g. acquiring tablets, changes of time allocated for sessions)
- What long term effects of the intervention would you consider important in order to recommend further implementation of DIALOG+?
- What profile of mental health professional is most likely to adopt DIALOG+ in their clinical practice?
- What profile of mental health patients is most likely to participate in DIALOG+ sessions?
- How would you promote the intervention in your country?

**5) End of interview**

Is there anything you would like to add? Any final thoughts or ideas?

Thank you very much for your time. Your opinions will be a valuable asset to the study.

*Remind participant that all information is confidential.*

# Appendix B- topic guide for in-depth semi-structured interviews with patients

**Experiences of DIALOG+ intervention
[patient participants]**

*Topic guide for interviews with patients who used the DIALOG+ intervention*

**To obtain and explore ideas of:**

- Patients’ experiences of using the DIALOG+ intervention
- Impact of DIALOG+ on patients life
- Views on collaborative working between clinicians and patients
- Views on scaling up the DIALOG+ intervention and its sustainability in the local context
- Suggested improvements of using the intervention

**INTRODUCTION (7 min)**

- **Thank participant for their availability and introduce yourself and other researchers**
  - *I am XXX. I am a researcher with the IMPULSE study, and I will ask you some questions about your experiences with using the DIALOG+ intervention.*
- **Explain nature and purpose of the research and provide a reminder of DIALOG+ (show the tablet with DIALOG+ app)**
  - *You were a part of the IMPULSE study. The purpose of this study is to test a new intervention called DIALOG+ which involved using a tablet with the DIALOG+ application in the routine meetings between you and your clinician.*
  - *DIALOG+ is an app-based therapeutic approach which aims to improve the communication between clinicians and patients and, through that, improve patients’ wellbeing. In DIALOG+, patients use a tablet to complete the DIALOG scale in the meeting with their clinicians. Based on the ratings and comparisons with previous ratings, patients decide which concerns they want to discuss in the given meeting. Each concern is then addressed with the clinician, and then the patient and clinician agree on actions/activities which will then be reviewed at the next meeting.*
- **Explain the purpose of the interview**
  - *The purpose of this interview is to find out more about what you experienced during the DIALOG+ sessions.*
- **Provide assurances about confidentiality**
  - *Your name and information will only be known by the researchers and will not be revealed to anyone else.*
- Inform participant the **session will be recorded** and introduce tape recorder.
- **Explain what happens to data collected** – transcribing, reported, anonymity- check how they would like to be referred to during recording
  - *The session will be audio-recorded, transcribed and analysed by the researchers and audio recordings will be destroyed once data analysis is complete. For any quotes that we use you will be identified by an ID number and all potentially identifying information will be removed.*
- **Explain how the interview works** – interviewer will not say very much and will tend to ask questions, your views are important, no right or wrong answers.
- **Explain they should say if they are feeling stressed or uncomfortable** and that they do not have to answer a question and that a topic can be changed if they need to. The interview can be paused for a break or stopped at any time if you no longer want to continue.
- **Show short video of an example interview**
- Discussion can last up to 1.0h
- Mobile phones are off or on silent.
- Invite any questions before you begin.

**NOTES FOR THE INTERVIEWER:**

- For the following questions, it is not necessary to stick to the exact line of questioning or order. The purpose of these questions is to be open and explorative. Topics to start off with an open discussion, and then use prompts to guide the discussion.
- It is important, however, that the subject matters in the topic guides are explored and the style of questioning kept open and explorative. Not leading.
- The interviewer has a responsibility to adequately cover all prepared questions within the time allotted. S/he also has a responsibility to get the participant to talk and fully explain their answers. Some helpful general probes include:
  - *“Can you tell me more about that?”*
  - *“What is it like?”*
  - *“What led you to (feel, think in specific way). . . “*
  - *“Can you think of another example of this?”*
  - *“Can you give me more details please?”*
  - *“I’d like to hear more about…”*
  - *“How did that make you feel?”*
  - *“Can you help me understand what you mean…”*

**BACKGROUND (3 min)**

Ask participant to introduce themselves and say a little bit about themselves. Interviewer to begin.

**TO EXPLORE PATIENT’S EXPERIENCE OF USING DIALOG+ (20 min)**

1. For the last year or so, as part of this study, you started using a tablet to discuss a set of topics during your meetings with your clinician. This intervention is called DIALOG+. **Can you talk me through how you and your clinician would use this and what happened during the sessions?**

***Probes:***

- *How easy or difficult did you find to participate in the DIALOG+ sessions? What made it easy/difficult?*
  - *Did your mental of physical health prevent you from attending/participating the sessions in any way?*
  - *How easy or difficult did you find using the tablet during sessions?*
  - *Did DIALOG+ cause you any discomfort?*
  - *If you had difficulties with participating in DIALOG+, how did you overcome them?*
- *How much did you like or dislike conversations you had in DIALOG+ sessions?*
- *Were DIALOG+ sessions too short or too long for you?*
- What did you think about having 6 DIALOG+ sessions over 12 months? Would you have liked to do it more or less often?
- *How confident did you feel about participating in DIALOG+ sessions?*
- *Were family members/carers involved in your DIALOG+ sessions? Was this helpful/useful?*
- *Considering your responses, overall, how appropriate did you feel these DIALOG+ sessions were for you?*

1. **How did your experience change over time? (compare 5-6^th^ session with 1-2 session, change for the better or worse)**

**TO EXPLORE THE IMPACT OF DIALOG+ ON PATIENTS LIFE (5 min)**

1. **How do you think participating in the DIALOG+ sessions has affected you?**

***Probes:***

- *How has your overall situation changed, if at all? In what ways has it improved/not improved?*
- How helpful/useful was participating in the DIALOG+ sessions?
- *Did family members/carers notice any change?*

**TO EXPLORE THE COLLABORATIVE WAY OF WORKING BETWEEN PATIENTS AND CLINICIANS (10 min)**

1. The DIALOG+ sessions may have been different from the usual meetings with your clinician/psychiatrist. **What was the same and what was different for you, compared to your meetings in the past?**

***Probes:***

- *Were you asked to choose topics for discussion in DIALOG+ sessions?*
- *Who would decide on activities/homework, you or/and your clinicians? How did you feel about that?*
- *Would you have liked the topics and/or activities to be chosen in a different way? (e.g. you alone, clinician alone or a joint decision)*
- *Do you think you were seen as a partner in these discussions?*

1. At the end of each session, you and your clinician set actions/activities/homework to be completed before the next session. **What did you like or dislike about setting and completing actions?**

***Probes:***

- *What was easy or difficult about setting the actions?*
- *Do you feel you were able to complete the actions set before your next DIALOG+ session? What was easy or difficult about completing the actions?*
- *Did your family members support you in completing actions? In what way?*
- *Did you use the patient booklet in setting and completing actions? Did you find it useful? Why/why not?*
- *Did you and your clinician talk about the actions from the previous session at the beginning of the next session?*
- *What actions, if any, were important to you?*

**TO EXPLORE SCALING UP AND SUSTAINABILITY OF DIALOG+ (5 min)**

1. **Would you like to continue using the DIALOG+ intervention? Why/why not?**

***Probes:***

- *Do you think the DIALOG+ intervention would be useful for other patients? Why/why not?*
- *Do you think that the DIALOG+ intervention should be offered to patients as part of the clinical care in this country? Why/why not? If yes, how can that happen*

**TO EXPLORE SUGGESTED IMPROVEMENTS OF USING DIALOG+ (5 min)**So far we discussed DIALOG+ or the intervention that uses the tablet, which you had a chance to try out. At the end let’s speak about what needs to be improved.

1. **If we were to offer this intervention to other patients, could you please suggest what would we need to improve so that other patients could also successfully use it?**

**CLOSING REMARKS (5 min)**

Do you have anything else to add to what we have discussed today: any further comments or recommendations?

Thank you for your contribution.

*Remind participant that all information is confidential*

# Appendix C- topic guide for in-depth semi-structured interviews with clinicians

**Experiences of DIALOG+ intervention
[clinician participants]**

*Topic guide for interviews with clinicians who delivered the DIALOG+ intervention*

**To obtain and explore ideas of:**

- To assess the fidelity of the intervention delivery
- To explore perceived usefulness/effectiveness
- To explore the collaborative way of working between patients and clinicians (novelty of the intervention)
- To explore the sustainability of the intervention and how it could be rolled-out in clinical settings if shown to be effective

**INTRODUCTION (10 min)**

- **Thank participants for their availability and introduce yourself and other researchers**
  - *I am XXX. I am a researcher with the IMPULSE study, and I will ask you some questions about your experiences with this study.*
- **Explain nature and purpose of the research and provide a reminder of DIALOG+**
  - *You were a part of the IMPULSE study. The purpose of this study is to test DIALOG+ in the routine meetings between you and your patient.* *DIALOG+ is an app-based therapeutic approach which aims to improve the communication between clinicians and patients and, through that, improve patients’ wellbeing...*
- **Explain the purpose of the interview**
  - *The purpose of this interview is to find out more about what you experienced during the DIALOG+ sessions.*
- **Provide assurances about confidentiality**
  - *Your name and information will only be known by the researchers and will not be revealed to anyone else.*
- Inform participants the **session will be recorded** and introduce tape recorder.
- **Explain what happens to data collected** – transcribing, reported, anonymity- check how they would like to be referred to during recording
  - *The session will be audio-recorded, transcribed and analysed by the researchers and audio recordings will be destroyed once data analysis is complete. For any quotes that we use you will be identified by an ID number and all potentially identifying information will be removed.*
- **Explain how the interview works** – interviewer will not say very much and will tend to ask questions, your views are important, no right or wrong answers.
- **Explain they should say if they are feeling stressed or uncomfortable** and that they do not have to answer a question and that a topic can be changed if they need to. The interview can be paused for a break or stopped at any time if you no longer want to continue.
- Discussion can last up to 1.0h
- Mobile phones are off or on silent.
- Invite any questions before you begin.

**NOTES FOR THE INTERVIEWER:**

- For the following questions, it is not necessary to stick to the exact line of questioning or order. The purpose of these questions is to be open and explorative. Topics to start off with an open discussion, and then use prompts to guide the discussion.
- It is important, however, that the subject matters in the topic guides are explored and the style of questioning kept open and explorative. Not leading.
- The interviewer has a responsibility to adequately cover all prepared questions within the time allotted. S/he also has a responsibility to get all participants to talk and fully explain their answers. Some helpful general probes include:
  - *“Can you tell me more about that?”*
  - *“What is it like?”*
  - *“What led you to (feel, think in specific way). . . “*
  - *“Can you think of another example of this?”*
  - *“Can you give me more details please?”*
  - *“I’d like to hear more about…”*
  - *“How did that make you feel?”*
  - *“Can you help me understand what you mean…”*

**BACKGROUND (3 min)**

Ask the participant to introduce themselves and say a little bit about themselves.

**TO ASSESS THE FIDELITY OF THE INTERVENTION DELIVERY (20 -25 min)**

1. For the last year or so you have been delivering the DIALOG+ intervention to patients. **Can you talk me through how you and your patients would use DIALOG+ and what happened during the sessions?**

***Probes:***

- *How easy or difficult did you find it to deliver the DIALOG+ intervention? What made it easy/difficult? Is there anything that would make it easier?*
  - *Please consider what was easy or difficult in regards to patients, clinicians and services involved in the intervention?*
  - *Were there any patients that DIALOG+ did not work well with?*
  - *What do you think of the training and supervision you received from researchers during the study?*
  - *What kind of support did you have from your service manager/line manager?*
  - *If you had difficulties, how did you overcome them?*
- *How did you find conversations you had with your patients in DIALOG+ sessions?*
- *How did you manage the time it took to deliver DIALOG+? Were DIALOG + sessions too short or too long?*
- *How did you find using a tablet computer in your work with patients?*
- *How confident did you feel about delivering DIALOG+?*
- How did your experience change over time?

**TO EXPOLORE PERCEIVED USEFULNESS/EFFECTIVENESS (15 - 17 min)**

1. **Do you think there have been benefits, if any, to the use of the DIALOG+ intervention? If yes, what were they and how were they achieved?**

***Probes:***

- - *What changes have you noticed in your patients’ behaviour and communication? What do you think has caused these changes?*
  - *Have your practice or service improved in any way?*
  - *Considering your responses, overall, how appropriate do you think these DIALOG+ sessions were for your practice?*
  - *What did not work well in the Dialog+ intervention?*

**TO EXPLORE THE COLLABORATIVE WAY OF WORKING BETWEEN PATIENTS AND CLINICIANS (20 min)**

1. The DIALOG+ sessions may have been different from your usual routine meetings with patients. **How did the DIALOG+ approach change your communication with your patients included in the intervention arm of the trial?**

***Probes:***

- *How did using a tablet and DIALOG+ impact your therapeutic relationship?*
- *Do you think your patients became more proactive in discussions?*
  - *Were DIALOG+ sessions more patient-led as compared to before?*

1. At the end of each session, you and your patients set actions/activities/homework to be completed before the next session. **How did you go about setting actions?**

***Probes:***

- *Did you review action items from a previous session?*
- *Do you feel that the actions agreed were mutually agreed between you and the patients?*
- *What was easy/difficult about setting the actions?*
  - *What would make it easier to set actions? Is there anything that helped you?*

**TO EXPLORE THE SUSTAINABILITY OF THE INTERVENTION (10 min)**

1. **Would you like to continue using DIALOG+ as part of your routine clinical practice? Why/why not?**

***Probes:***

- *Do you think that the DIALOG+ intervention should become part of the clinical practice in this country?*
- *What do you think needs to change in services (service provision, clinicians) for the DIALOG+ intervention to become part of the clinical practice?*

1. **If you had to suggest just one key improvement for other clinicians to successfully deliver DIALOG+ in their routine clinical meetings what would you say**?

**CLOSING REMARKS (5 min)**

Do you have anything else to add to what we have discussed today: any further comments or recommendations?

Thank you for your contribution.

*Remind the participant that all information is confidential*
